# Spectrophotometric Determination of Ca^2+^ and Ca-Complex Formation Constants: Application to Chemical Enhanced Oil Recovery

**DOI:** 10.1021/acsomega.0c06185

**Published:** 2021-02-05

**Authors:** Dirk J. Groenendijk, Ron Bouwmeester, Johannes N. M. van Wunnik

**Affiliations:** Shell Global Solutions International B.V., Amsterdam, Grasweg 31, 1031 HW Amsterdam, The Netherlands

## Abstract

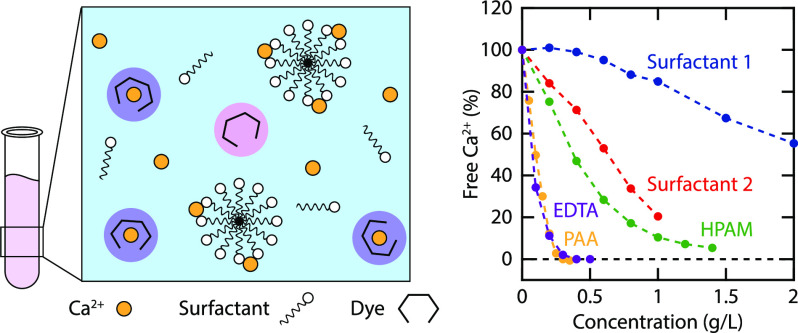

Chemicals such as anionic surfactants and polymers often contain groups that complex divalent ions such as Ca^2+^. The formation of divalent ion complexes can decrease emulsifying or viscosifying power and lead to adsorption or precipitation. This is particularly relevant in chemical enhanced oil recovery, where high viscosities and low interfacial tensions are required for mobility control and the formation of oil–water microemulsions, respectively. In this work, we use a Ca^2+^-sensitive dye to determine the Ca^2+^ concentration and Ca-complex formation constants in solutions containing complexing agents. This method can be used to rapidly screen the affinity of different chemicals to form Ca-complexes in low-salinity solutions. The complex formation constants can be implemented into chemical flooding simulators to investigate the interplay with mineral dissolution and cation exchange and model adsorption processes.

## Introduction

Chemical enhanced oil recovery (EOR) relies on the injection of chemicals such as polymers and surfactants to improve oil recovery.^1−3^ Polymers are added to the waterflood to increase the viscosity of the injection water and improve the mobility ratio, resulting in an increase in the sweep efficiency.^4^ Surfactants are added to reduce the oil–water interfacial tension, resulting in the release of capillary trapped oil.^5^ The effectiveness of these chemicals depends on the reservoir mineralogy, temperature, heterogeneity, and ionic environment and is particularly sensitive to the concentration of divalent ions. Complexation of divalent ions such as Ca^2+^ and Mg^2+^ lowers the viscosity of polymer solutions by introducing attractive polymer–polymer interactions and leads to precipitation at high concentrations.^6,7^ Surfactants also tend to precipitate at high divalent ion concentrations, resulting in an increase in the interfacial tension.^8^ Divalent ions can also mediate the adsorption of anionic surfactants and polymers to negatively charged rock surfaces through a process called cation-bridging, resulting in higher concentrations required for chemical flooding.^9−12^ It is therefore highly important to measure the affinity of different chemicals to interact with divalent ions and determine the formation constants of their complexation reactions.

In the following, we consider the complexation of divalent ions by anionic surfactants. Above the critical micelle concentration (CMC), most surfactant monomers are incorporated in micelles to minimize the contact of the hydrophobic tails with the water phase. The negative charge on the micelle surface is screened by attractive electrostatic interactions with the cations in solution. In addition, the surfactant headgroups (e.g., carboxylate, sulfate, or sulfonate groups) can form complexes with divalent ions such as Ca^2+^.^13,14^ The micelles with complexed Ca^2+^ ions remain in solution at low Ca^2+^ concentrations, while the surfactant can precipitate as Surf_2_Ca at high concentrations. Complexation also leads to the somewhat counterintuitive observation that the Ca^2+^ tolerance of the surfactant solution increases with increasing surfactant concentration, caused by the effective decrease in [Ca^2+^] in solution. The complexation of Ca^2+^ by anionic surfactants can be expressed as
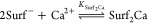
1where
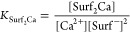
2

Note that this equation also applies to Ca^2+^ complexation by surfactants that are part of a micelle (when the charge of two neighboring surfactant headgroups is neutralized by a Ca^2+^ ion). Given that
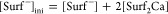
3and
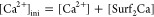
4where “ini” refers to the initial concentration, *K*_Surf_2_Ca_ can be expressed as
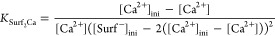
5

Since [Surf^–^]_ini_ and [Ca^2+^]_ini_ are known, only the free (or bound) Ca^2+^ concentration is required to calculate the complex formation constant. Expressions for the formation constants of other chemicals can be derived in the same manner. However, measuring the unbound Ca^2+^ concentration in solutions with complexing agents is not straightforward: methods relying on Ca^2+^-selective electrodes, dialysis membranes, and activated carbon columns were explored, but all presented complications such as fouling, long diffusion times, and possible dissociation of Ca-complexes when the solution passes through the column. We found the use of a Ca^2+^-sensitive dye (Pontachrome Violet SW) to be the most effective method to measure the free Ca^2+^ concentration in aqueous solutions. This dye exhibits a change in absorption spectrum upon forming a complex with Ca^2+^ and has previously been used to measure the free Ca^2+^ concentration in detergent formulations.^15^ The chemical structure of Pontachrome Violet SW is shown in Figure 2 (inset). It is suited to measure Ca^2+^ concentrations in the order of tens of mg/L due to its small formation constant and can be used at pH values between 9.5 and 11, where its extinction coefficient ε_a1_ remains constant.

**Figure 1:**
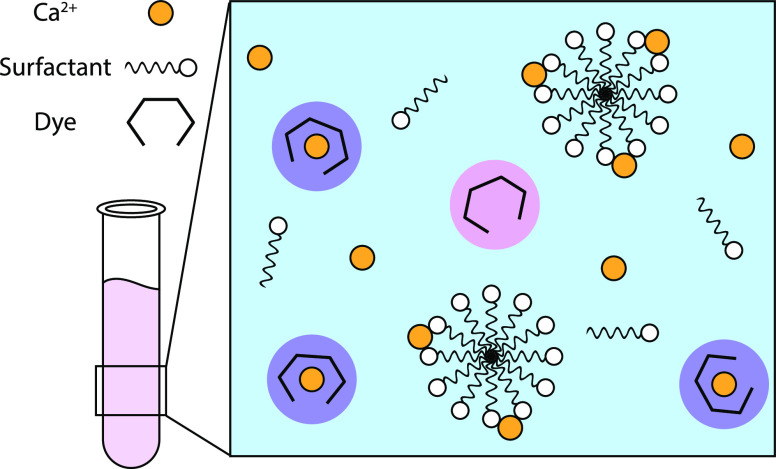
Schematic representation of the solution containing Ca^2+^, surfactant and dye. Na^+^ ions are omitted for clarity.

Here, this method is used to measure the Ca^2+^-complexing potential of different chemicals: two surfactants (ENORDET J771 and ENORDET O332), sodium polyacrylate, hydrolyzed polyacrylamide (HPAM), and ethylenediaminetetraacetic acid (EDTA). The results apply to solutions without Mg^2+^ ions and with relatively low salinity values, which is relevant for a subset of oil and gas reservoirs. A schematic representation of the solution containing Ca^2+^ surfactant, and dye is shown in Figure 1, illustrating the change in absorbance of the dye upon binding Ca^2+^.

## Results and Discussion

The addition of the dye (D^2–^) to deionized (DI) water containing Ca^2+^ ions introduces the following equilibrium:
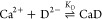
6where
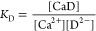
7

The absorption spectrum of a single dye molecule changes upon forming a complex with Ca^2+^ (CaD). Therefore, the absorbance of a solution containing many dye molecules will be the average of the bound and unbound dye molecules. By using a fixed initial dye concentration (D_0_) and knowing *K*_D_, the absorbance of the solution can be measured with ultraviolet–visible (UV–Vis) spectroscopy to determine [CaD]/[D^2–^] and the free Ca^2+^ concentration ([Ca^2+^]). A relation between the absorbance and [Ca^2+^] can be obtained as follows. Let *y* be the molar fraction of free dye such that [D^2–^] = D_0_ · *y*. Then
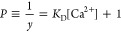
8

According to Lambert–Beer’s law
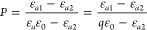
9

where ε*_a_* is the molar extinction coefficient of the sample solution and ε_*a*1_ = 8.87 × 10^3^ cmL/mol, ε_*a*2_ = 0.864 × 10^3^ cmL/mol, and ε_0_ = 7.74 × 10^3^ cmL/mol are the molar extinction coefficients for the unbound dye, dye–calcium complex, and isosbestic point (520 nm), respectively. *q* = ε*_a_*/ε_0_ can be obtained by measuring the absorbance at *λ* = 520, 575, and 680 nm:
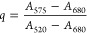
10

**Figure 2:**
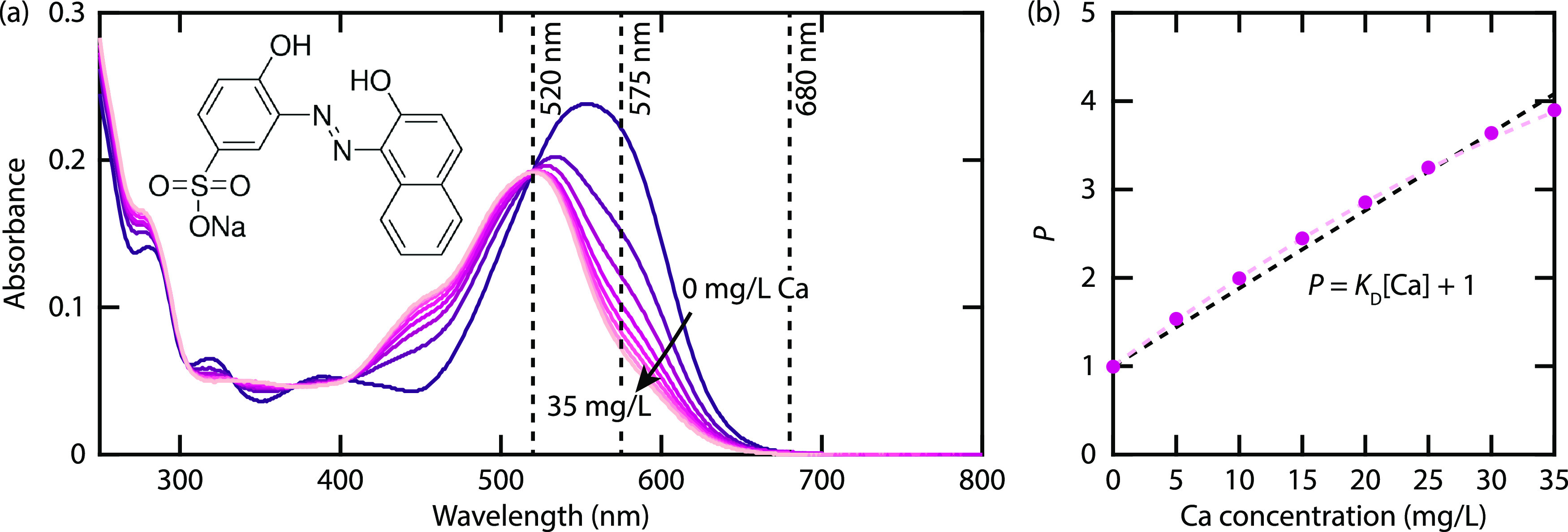
(a) Absorption spectra of solutions containing 10 mg/L dye as a function of [Ca], in steps of 5 mg/L. (b) Parameter *P* as a function of the Ca concentration. The gray line is a linear fit passing through (0,1).

The absorbance at 520 nm is used as a reference point because this is the isosbestic point (the absorbance at this wavelength is constant), while the absorbance at 680 nm is subtracted to remove any background signal due to, for example, system drift. The absorbance at 575 nm is used because, at that wavelength, it is the most sensitive to the Ca^2+^ concentration. The first equation shows that *P*([Ca^2+^]) is a straight line passing through (0,1) with slope *K*_D_. Therefore, *K*_D_ can be determined by measuring the absorbance as a function of [Ca]. The measured spectra are shown in Figure 2a for [Ca^2+^] ranging from 0 to 35 mg/L in steps of 5 mg/L.

From the measured absorbance data, we can calculate *P* using eqs 9 and 10, which is shown as a function of [Ca] in Figure 2b. Fitting the data (with an intersection at (0,1)) yields *K*_D_ = 0.0881. We note that a second-order polynomial provides a slightly better fit (pink dashed line) but the difference is minimal, and this would require a refinement of eq 7.

Having determined *K*_D_, we can determine the free Ca^2+^ concentration in solutions containing complexing agents simply by measuring the absorbance and using the relation [Ca^2+^] = (*P* – 1)/*K*_D_. In all experiments, the same initial amount of dye (*D*_0_ = 10 mg/L) was used and 140 mg/L NH_4_OH was added to increase the pH to approximately 10.5. Before applying this method, we addressed to what extent the spectra are affected by time, salinity, and the possible interaction with the complexing agents. Several experiments were performed for this purpose, which are included in the Supporting Information. This led to the following observations and constraints. (i) The absorbance slowly increases over time, likely caused by a change in pH due to CO_2_ dissolution. To take this into account, the calibration and subsequent measurements were performed within 30 min after the preparation of the solutions. (ii) Higher NaCl concentrations result in lower measured Ca concentrations by competitive binding and by modifying the activities (*γ*). The calibration curve in DI water is therefore no longer applicable when measuring in solutions with higher salinities. While it is possible to do the calibration at a given salinity, all UV–Vis measurements in this report were performed in DI water. Note that the solution cannot contain any Mg since the MgD complex is 100 times more stable than the CaD complex. (iii) Measurements with and without the Ca-complexing agents indicated that they do not interfere with the dye: in all cases, the absorbance at *λ* > 350 nm was equal when no Ca^2+^ was present in solution. The spectrophotometric method, which relies on the absorbances at 520, 575, and 680 nm, can therefore be applied to determine Ca^2+^ in solutions containing these agents.

**Figure 3:**
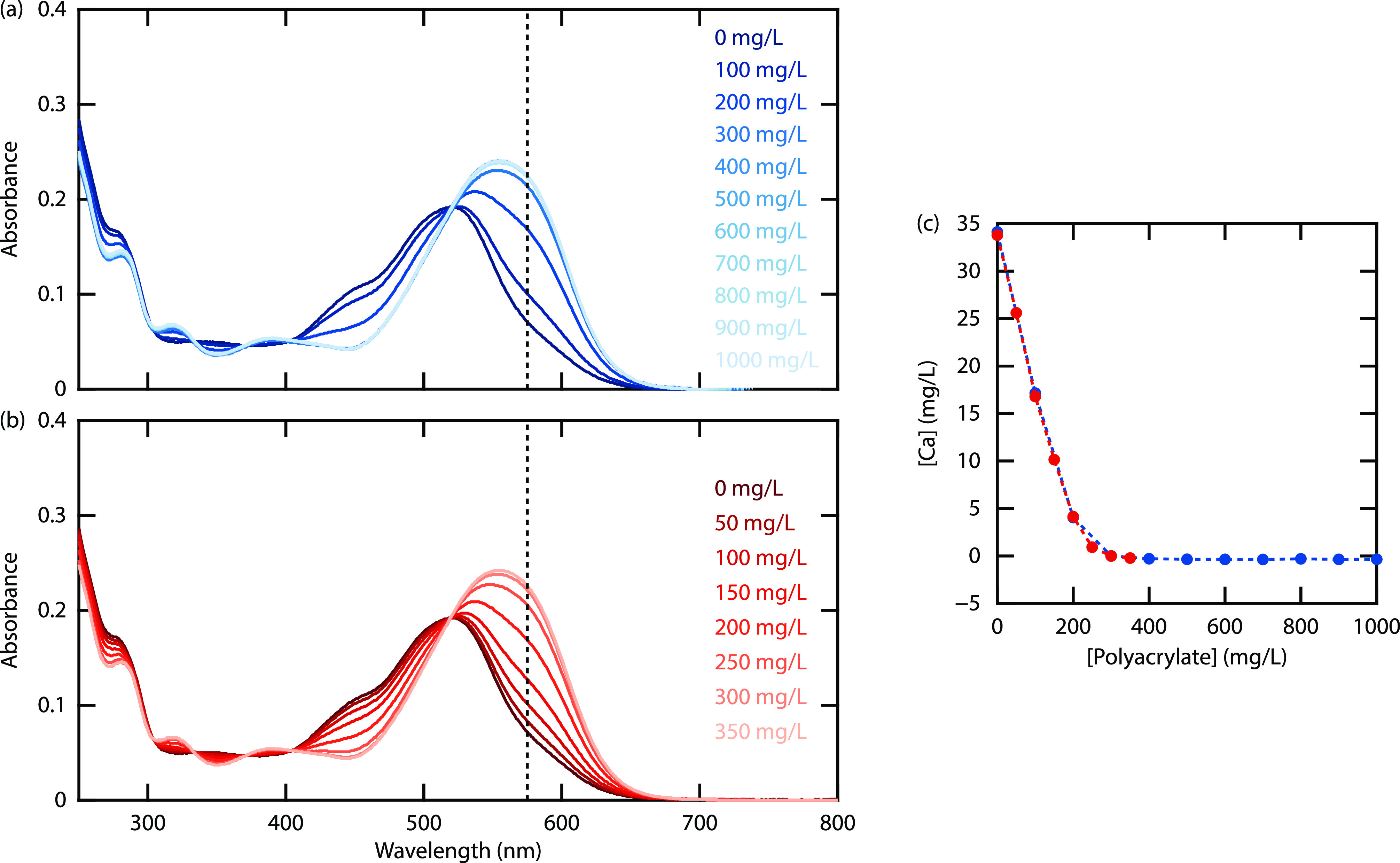
(a, b) Absorption spectra of solutions containing DI water and different concentrations of sodium polyacrylate. In (a) and (b), the concentrations are varied in steps of 100 and 50 mg/L, respectively. (c) [Ca] as a function of [polyacrylate]. Red and blue dots refer to the fine and coarse scans in concentration, respectively.

We first consider the affinity of an acrylic acid homopolymer (Flosperse 3000, average molecular weight = 4500 Da), which we will refer to as polyacrylate (PAA), to complex Ca^2+^. At a pH value of 10.5, the carboxylic acids groups (−COOH) are fully deprotonated, and the resulting carboxylate groups (−COO^–^) have a high affinity to complex divalent ions. These groups are also present in common polymers and surfactants used in chemical EOR applications. The absorption spectra of solutions with different PAA concentrations are shown in Figure 3. Two sets of measurements were performed with concentration increments of 100 and 50 mg/L, shown in Figure 3a and Figure 3b, respectively. The corresponding free Ca^2+^ concentration is shown in Figure 3c.

**Figure 4:**
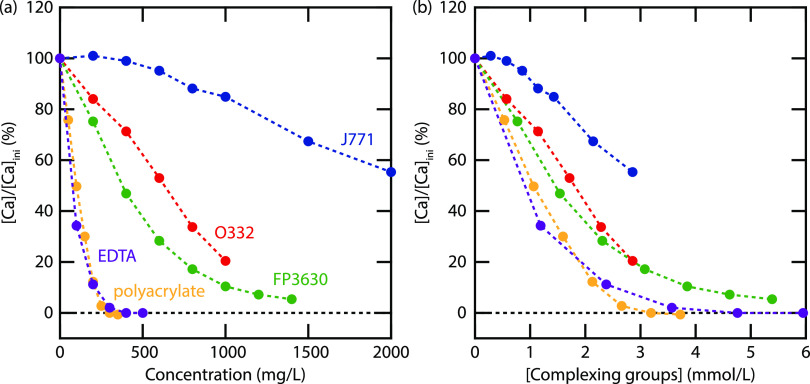
(a) Normalized [Ca^2+^] as a function of the additive concentration in mg/L. (b) Normalized [Ca^2+^] as a function of the number of chemical groups that can complex Ca^2+^.

The initial free Ca^2+^ concentration (35 mg/L) rapidly drops with increasing concentrations of polyacrylate, reaching zero at about 300 mg/L. The overlap of the two measurement runs shows that the results are highly reproducible. While large polymers tend to precipitate when binding Ca^2+^, the molecular weight of the polyacrylate is sufficiently low that it does not easily become insoluble and precipitate. The results for the other chemicals (EDTA, HPAM, and two surfactants) are shown in Figure 4a. The corresponding absorption spectra are included in the Supporting Information. Note that O332 surfactant is an internal olefin sulfonate (IOS) type surfactant, while J771 is an alcohol alkoxy sulfate (AAS) type surfactant. The investigated chemicals therefore constitute three different complexing groups: carboxylate (EDTA, PAA, and HPAM), sulfonate (O332), and sulfate (J771).

All investigated chemicals are found to lower the Ca^2+^ concentration. The affinity of polyacrylate to complex Ca^2+^ is roughly equal to that of EDTA, which is known for its high Ca^2+^-complexing potential and is even used to remove scale deposits. HPAM has a lower affinity to complex Ca^2+^ as it consists of both acrylamide (AM) and acrylic acid (AA). The two surfactants also lower the Ca^2+^ concentration, O332 being more effective than J771. No precipitation was observed in the experiments, and thus the decrease in [Ca^2+^] can be attributed to ion complexation.

The chemicals were compared on a weight basis (mg/L) in Figure 4a, which is a useful metric when considering how much of a certain chemical is required to complex a certain amount of Ca^2+^. To evaluate the complexing affinity of different chemical groups, it is more insightful to compare them by the number of Ca^2+^-complexing groups. In Figure 4b, the concentration has been rescaled to represent the number of chemical groups that can complex Ca^2+^ (in mmol/L). For PAA, O332, and J771, molecular weights of 94, 350, and 700 Da were used. Note that the molecular weight of PAA includes both the acrylate group and the Na^+^ counterion. Disodium EDTA has a molecular weight of 336.2 Da but contains four carboxylate groups, so a molecular weight of 84.05 Da was used in calculating the number of complexing groups. The HPAM polymer (FP3630, molecular weight of approximately 20 million Da) is often used to increase the water viscosity in polymer flooding and consists of AM and AA groups with molecular weights of 71 and 94 g/mol, respectively. The polymer has a hydrolysis degree of about 30%, meaning that 30% of the monomers are in the form of acrylic acid. From this, we can calculate that 1000 mg/L polymer contains 362 mg/L (3.85 mmol/L) acrylic acid groups, which are able to bind Ca^2+^ ions. To rescale the concentrations, an effective molecular weight of 260 Da (1000 mg/L/3.85 mmol/L) was therefore used (see the Supporting Information). Note that an active matter of 100% was used in this calculation. The resulting plot in Figure 4b shows that the curves of EDTA, polyacrylate, HPAM, and O332 are bunched together, indicating that their affinities to complex Ca^2+^ are nearly the same. The curve of J771 is shifted to the right, reflecting the lower affinity of the sulfate () group to complex Ca^2+^ than the carboxylate (COO^–^) and sulfonate () groups. Part of the differences can likely also be attributed to uncertainties in the hydrolysis degree of FP3630 or the active matter of the liquid emulsions.

The chemical modeling software PHREEQC is used to model the data in Figure 4 and determine the formation constants of their Ca^2+^-complexation reactions.^16^ We will demonstrate this for two chemicals, J771 and polyacrylate, considering the following possible reactions:
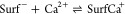
11
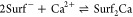
12
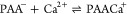
13
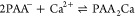
14

**Figure 5:**
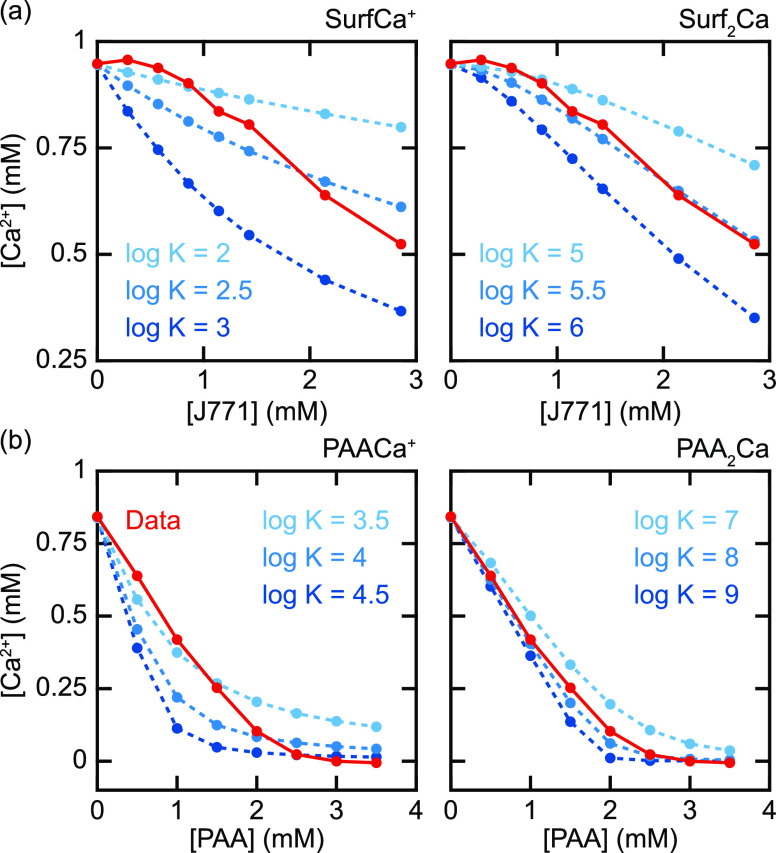
Simulated [Ca^2+^] versus [J771] and [PAA]. (a) SurfCa^+^ (left), Surf_2_Ca (right). (b) PAACa^+^ (left), PAA_2_Ca (right).

Note that the reactions with Surf_2_Ca and PAA_2_Ca do not necessarily imply that precipitates are formed; the reaction 2Surf^–^ + Ca^2+^ ⇌ Surf_2_Ca is equally valid to describe attachment of Ca^2+^ ions on surfactant micelles and can also be defined for *n* monomers and *n*/2 Ca^2+^ ions. The reactions may also be different in the presence of a negatively charged surface, in which case a SurfCa^+^ complex may adsorb to the surface. For each reaction, the formation constant is varied and the solution composition at equilibrium is calculated. The resulting Ca^2+^ concentrations are shown as a function of [J771] and [PAA] in Figure 5. The behavior closely resembles the data when considering reactions between one Ca^2+^ ion and two J771 or PAA molecules (when a neutral complex is formed). Good agreement is obtained with log *K* = 5.5 for the formation of Surf_2_Ca and log *K* = 8.0 for the formation of PAA_2_Ca. The equilibrium constant for the formation of EDTA–Ca^2+^ complexes determined using this method is approximately log *K* = 8.0, which is lower than the literature value of about 10.5.^17^ The lower value can likely be attributed to the formation of EDTA–Na^+^ and EDTA–H^+^ complexes at pH = 10.5.

**Figure 6:**
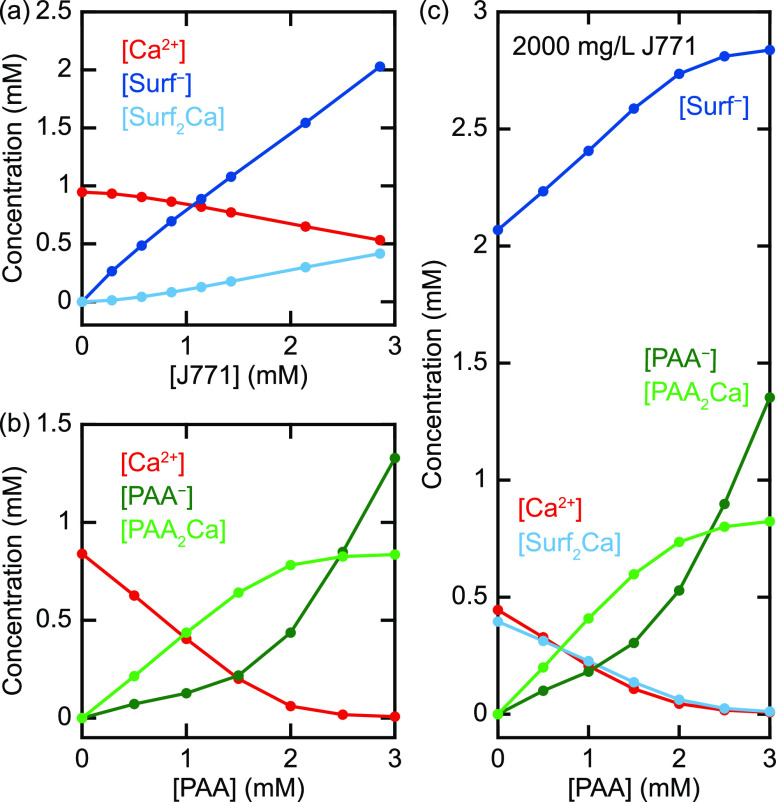
Calculated concentrations as a function of the total J771 (a) and PAA (b) concentration. (c) Calculated concentrations in a system containing both surfactant and PAA as a function of the total PAA concentration. 2000 mg/L J771 corresponds to 2.86 mmol/L.

For these reactions and formation constants, Figure 6a and Figure 6b show how the concentrations of the (un)complexed chemicals vary as a function of the total J771 and PAA concentrations, respectively. Figure 6b shows that the PAA_2_Ca concentration saturates when nearly all the Ca^2+^ is complexed by PAA. Having established both formation constants, we can calculate the concentrations in a system containing both surfactant and PAA. Figure 6c shows the Ca^2+^, Surf^–^, Surf_2_Ca, PAA^–^, and PAA_2_Ca concentrations in a solution containing 2000 mg/L (2.86 mM) J771 and different concentrations of polyacrylate. The results show that increasing the PAA concentration lowers the amount of Ca^2+^ complexed by the surfactant due to the higher affinity of PAA to complex Ca^2+^.

This points toward a potential strategy for preventing adsorption and precipitation of chemicals that are sensitive to Ca^2+^. This is particularly relevant for anionic surfactants, which readily adsorb to rock surfaces in the presence of divalent ions. In these cases, adding a chemical with a higher affinity to bind Ca^2+^ can lower adsorption by complexing the ions that are capable of mediating adsorption through cation bridging. The same mechanism could potentially be used to protect polymers, which suffer from a decrease in viscosity with increasing divalent ion concentration. Note that, for each application, the adsorption of the complex ingagent to the solid surface should also be evaluated.

## Conclusions

We showed that the Ca^2+^ concentration and Ca-complex formation constants in solutions containing complexing agents can be determined using a Ca^2+^-sensitive dye. The method can be used to rapidly evaluate the affinity of different chemicals to complex Ca^2+^, which can be useful in screening studies. The complex formation constants can be implemented into chemical flooding simulators, providing insight into the effectiveness of the chemicals during flooding. The spectrophotometric method can be extended to other research fields in which Ca^2+^ complexation is investigated, such as studies on cells containing bacteria and enzymes.

## Methods

All chemicals were used as received. Chemicals used were CaCl_2_·2H_2_O (Sigma Aldrich, ≥99.0%), NaCl (Sigma Aldrich, ≥99.0%), NH_4_OH (25%, Sigma Aldrich), Pontachrome Violet SW (Sigma Aldrich), EDTA (disodium salt dihydrate, Sigma Aldrich), sodium polyacrylate (Flosperse 3000, SNF), FP3630 (SNF), and ENORDET J771 and O332 (both developed by Shell^18,19^). ENORDET J771 is a C12–13–7 propoxy sulfate, APS; ENORDET O332 is a C15–C18, IOS. For deionized water, a Genie U Ultrapure & Reverse Osmosis water system (Rephile) was used (>18.0 MΩ at 25 °C). For the Ca-complexation experiments, solutions were prepared with 10 mg/L dye (Pontachrome Violet SW), 35 ppm Ca^2+^, 140 mg/L NH_4_OH, and different concentrations of the complexing agents. The solutions were shaken and shortly thereafter the absorption spectrum was measured with a UV–Vis spectrophotometer (Hach Lange, Model DR6000). The absorbance at 575 nm was used to determine the free Ca^2+^ concentration using the calibration curve in Figure 2.

## Supporting Information Available

Additional measurements focused on the applicability of the Ca-sensitive dye: (i) the time and salinity dependence of the spectra and (ii) the absorbance of the Ca-complexing agents. Absorption spectra of solutions containing Ca^2+^ and different concentrations of EDTA, FP3630, O332, and J771 are also included. The last section contains a calculation of the “effective” molecular weight of AA groups in HPAM (PDF).

## Abbreviations

AA: acrylic acid
AAS: alcohol alkoxy sulfate
AM: acrylamide
APS: alcohol propoxy sulfate
CMC: critical micelle concentration
DI: deionized
EDTA: ethylenediamine tetraacetic acid
EOR: enhanced oil recovery
HPAM: hydrolyzed polyacrylamide
IOS: internal olefin sulfonate
PAA: polyacrylic acid
UV-Vis: ultraviolet-visible
