# Supplementary material for: Spectrophotometric Determination of Ca2+ and Ca-Complex Formation Constants: Application to Chemical Enhanced Oil Recovery
Source: ACS Omega. 2021 Feb 5;6(7):5027–32. doi: 10.1021/acsomega.0c06185 (PMC7905941; doi:10.1021/acsomega.0c06185)
Supplement: Supplementary file 1 — ao0c06185_si_001.pdf [file ao0c06185_si_001.pdf]

Supporting Information for:
Spectrophotometric determination of Ca^{2+} and Ca-complex formation constants: application to chemical enhanced oil recovery

Dirk J. Groenendijk,[1,*] Ron Bouwmeester,[1] and Johannes N. M. van Wunnik[1]

[1]*Shell Global Solutions International B.V., Amsterdam, Grasweg 31, 1031 HW Amsterdam, The Netherlands*

Dated: January 22, 2021

CONTENTS

I. Absorbance vs. time S2

II. Absorbance vs. salinity S3

III. Absorbance of Ca-complexing agents S4

IV. Ca-complexation by different chemicals S5
- A. ENORDET J771 surfactant S5
- B. ENORDET O332 surfactant S5
- C. FP3630 polymer S6
- D. EDTA S6

V. Number of acrylic acid groups in HPAM S7

* dirk.groenendijk@shell.com

I. ABSORBANCE VS. TIME

It is important to evaluate to what extent time affects the spectra to ensure that reproducible results are obtained. This is depicted in Figure S1a, which shows absorption spectra of solutions containing 7.2 mg/L Ca measured at different times.

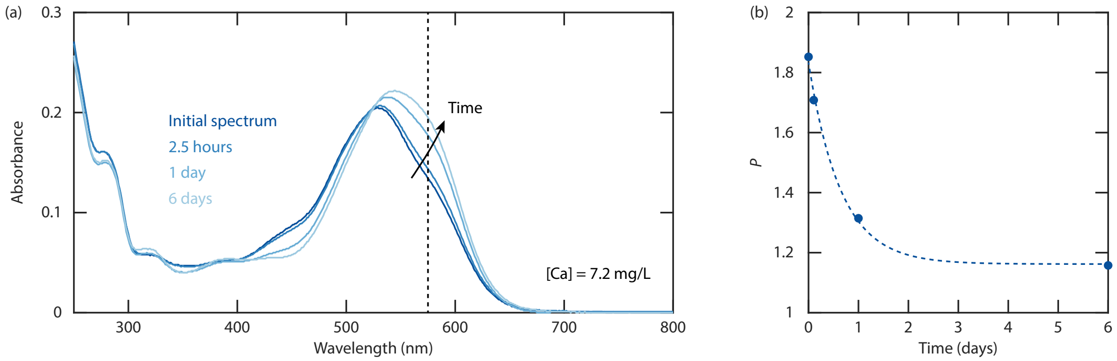

Figure S1: (a) Absorption spectra of a solution containing 10 mg/L dye and 7.2 mg/L Ca measured at different times. (b) P as a function of time.

Figure S1b shows how P varies as a function of time, decreasing relatively quickly and saturates after several days. This strongly affects the measured Ca concentration, which would appear to decrease by about 17% in 2.5 hours. The change of the absorption spectrum with time could be attributed to a decrease of the solution pH (due to CO_2 dissolution) or slow reactions affecting the dye. To make sure that all measured data is accurate, the calibration and subsequent measurements were performed within 30 minutes after the solutions were prepared.

II. ABSORBANCE VS. SALINITY

Salinity can affect the Ca–dye equilibrium by competitive complexation (e.g., formation of MgD in the case of Mg^{2+}) or by modifying the activities γ. Satsuki et al. report that the Mg-dye complex is 100 times more stable than the Ca-dye complex, therefore the solution should be free of Mg ions. Here, we consider the effect of added NaCl. Figure S2a shows absorption spectra as a function of salinity for [Ca] = 0 mg/L and [Ca] = 36 mg/L.

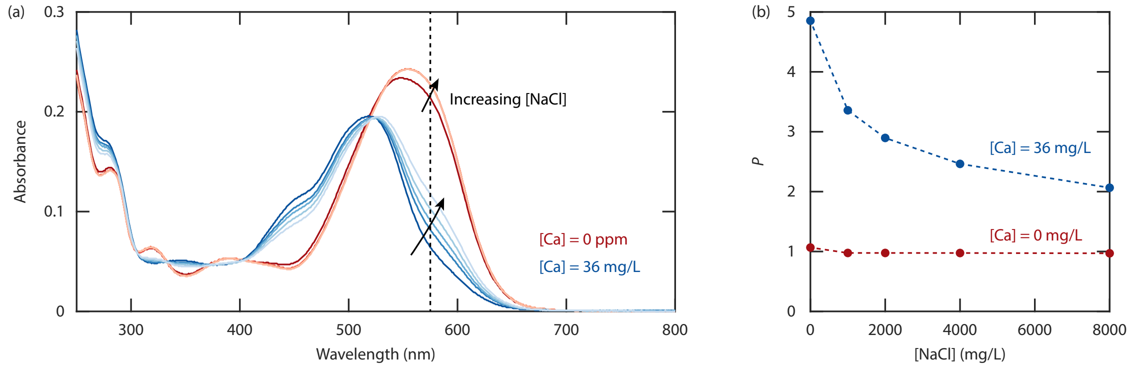

Figure S2: (a) Absorption spectra of solutions containing 10 mg/L dye without Ca (red lines) and with 36 mg/L Ca (blue lines) and NaCl concentrations from 0 to 8000 mg/L. (b) P as a function of [NaCl].

When [Ca] = 0 mg/L (red lines), the absorbance initially increases when 1000 mg/L NaCl is added. Adding more NaCl does not further increase the absorbance. However, when [Ca] = 36 mg/L (blue lines), the absorbance increases continuously with increasing [NaCl]. This can be seen in more detail in Figure S2b, where P is plotted as a function of [NaCl]. In the absence of Ca^{2+}, the only effect of the Na^+ and Cl^- ions on the dye molecule is to surround it and screen its charge, which slightly affects its absorption spectrum. Further increasing [NaCl] does not modify the spectrum, indicating that the 10 mg/L of dye molecules are saturated by Na^+ and Cl^-. In the presence of Ca^{2+}, however, there is competition between Na^+ and Ca^{2+} in complexation/screening, and the Na^+/Ca^{2+} ratio is continuously changing. Increasing NaCl concentrations therefore result in a lower measured [Ca], which has to be taken into account. This is also the reason that NH_4OH is used to increase the solution pH.

III. ABSORBANCE OF CA-COMPLEXING AGENTS

We now focus on whether the Ca-complexing agents affect the absorption spectrum of the dye. The spectrum of the dye should be identical with and without added agent, otherwise it is no longer possible to relate the absorbance to the free Ca concentration. Figure S3 shows absorption spectra of solutions containing 10 mg/L dye and (a) J771 surfactant, (b) O332 surfactant, (c) sodium polyacrylate, and (d) FP3630, each together with a measurement of a 'blank' solution (without additive). For the preparation of solutions containing J771, O332, and polyacrylate, active matters of 30.1%, 28.03%, and 42.7% were considered, respectively. All spectra are identical for $\lambda > 350$ nm, indicating that the Ca-complexing agents do not interfere with the dye. Our method, which relies on the absorbances at 520, 575, and 680 nm, can therefore be applied. The increased absorbance at low wavelengths, in particular of the solutions containing O332, FP3630 and EDTA, can be attributed to the absorption of these compounds.

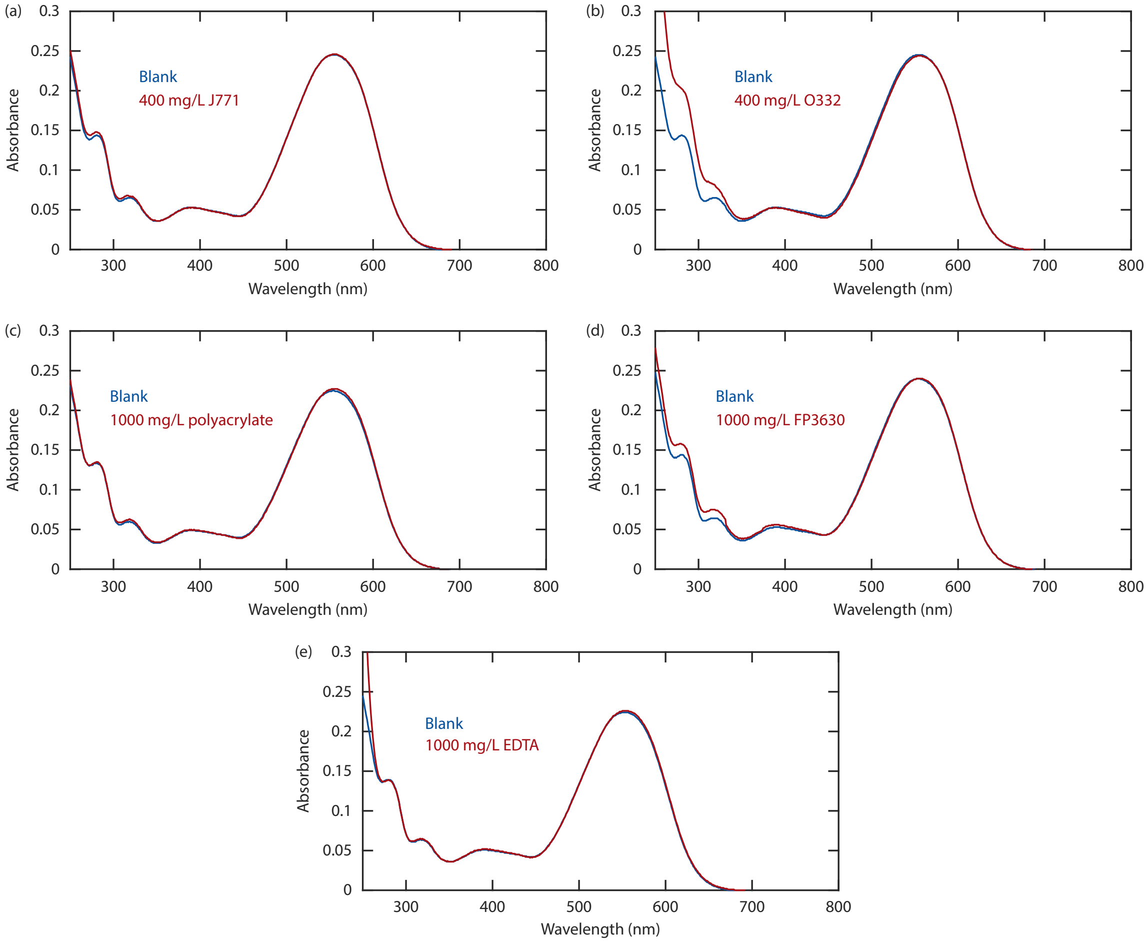

Figure S3: Absorption spectra of solutions containing 10 mg/L dye with and without Ca-complexing agents. (a) J771 surfactant, (b) O332 surfactant, (c) sodium polyacrylate (4500 Da), (d) FP3630 (20 million Da), and (e) EDTA.

IV. CA-COMPLEXATION BY DIFFERENT CHEMICALS

A. ENORDET J771 surfactant

The absorption spectra of solutions with different concentrations of J771 are shown in Figure S4a. The Ca^{2+} concentration as a function of [J771] is shown in Fig S4b.

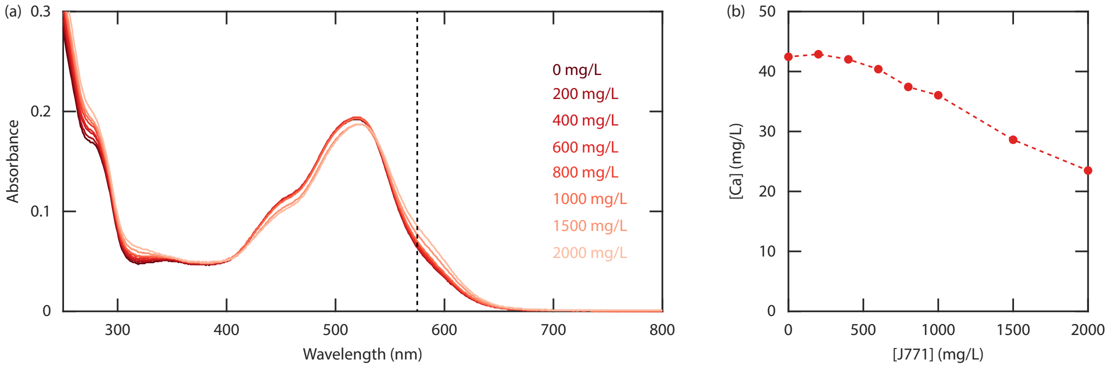

Figure S4: (a) Absorption spectra of solutions containing DI water and different concentrations of J771 surfactant. (b) $[Ca^{2+}]$ as a function of [J771].

B. ENORDET O332 surfactant

The absorption spectra of solutions with different concentrations of O332 are shown in Figure S5a. The Ca^{2+} concentration as a function of [O332] is shown in Fig S5b.

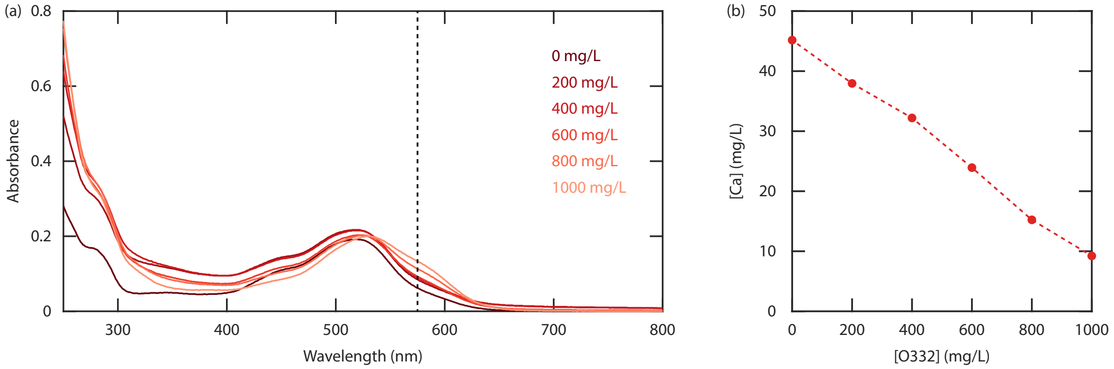

Figure S5: (a) Absorption spectra of solutions containing DI water and different concentrations of O332 surfactant. (b) $[Ca^{2+}]$ as a function of [O332].

C. FP3630 polymer

The absorption spectra of solutions with different concentrations of FP3630 are shown in Figure S6a. The Ca^{2+} concentration as a function of [FP3630] is shown in Fig S6b.

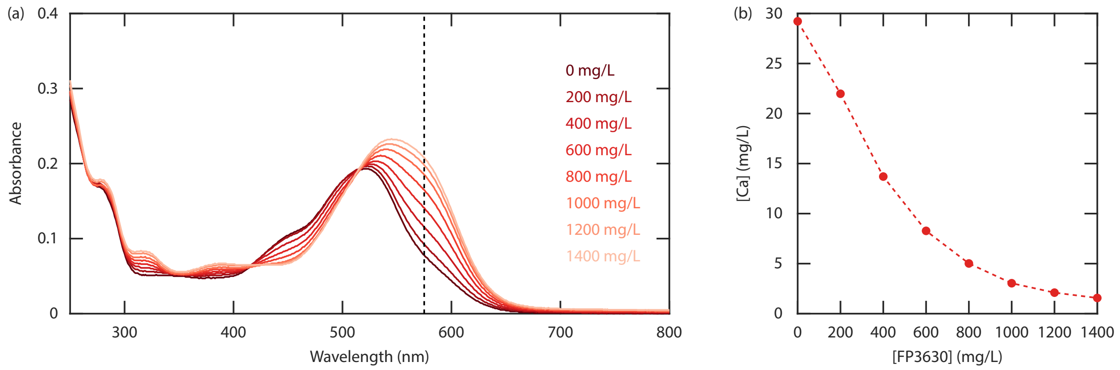

Figure S6: (a) Absorption spectra of solutions containing DI water and different concentrations of FP3630. (b) [Ca^{2+}] as a function of [FP3630].

D. EDTA

The absorption spectra of solutions with different concentrations of EDTA are shown in Figure S7a. The Ca^{2+} concentration as a function of [EDTA] is shown in Fig S7b.

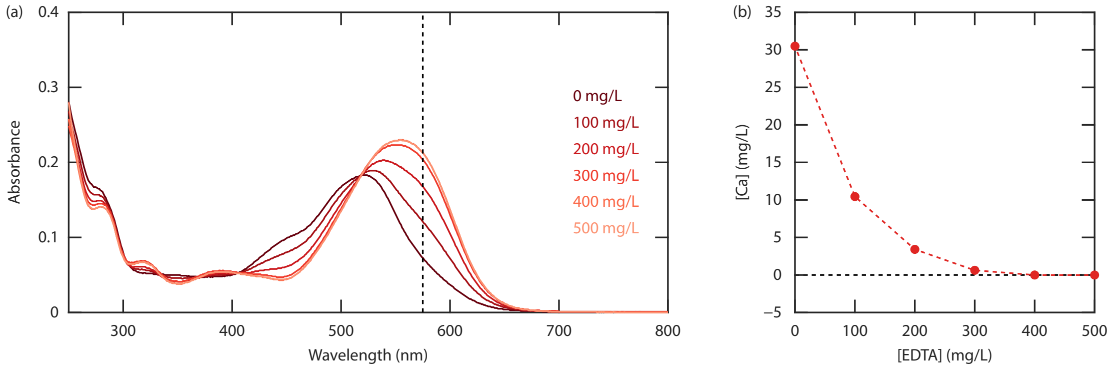

Figure S7: (a) Absorption spectra of solutions containing DI water and different concentrations of EDTA. (b) [Ca^{2+}] as a function of [EDTA].

V. NUMBER OF ACRYLIC ACID GROUPS IN HPAM

HPAM consists of AM and AA groups with molecular weights of 71 g/mol and 94 g/mol, respectively. The used polymer has a hydrolysis degree of about 30%, meaning that 30% (mole fraction) of the monomers are in the form of acrylic acid. This can be used to determine how many acrylic acid groups a certain amount of HPAM contains. We define the following:

$$x \text{ mg/L AM} = \frac{x}{71} \text{ mmol/L}, \quad y \text{ mg/L AA} = \frac{x}{94} \text{ mmol/L}, \tag{1}$$

The mole fraction of 30% AA can be expressed as:

$$\frac{x/71}{y/94} = \frac{70}{30}, \tag{2}$$

and if we have for example 1000 mg/L HPAM, we know that $x + y = 1000$ mg/L. These two equations can be solved for y, which yields 362 mg/L (3.85 mmol/L) AA groups. To convert the amount of HPAM in mg/L to mmol/L AA groups, we can therefore divide by the "effective" molecular weight:

$$\frac{1000 \text{ mg/L}}{3.85 \text{ mmol/L}} = 260 \text{ Da}. \tag{3}$$
